# Editorial: Newest challenges and advances in the treatment of colorectal disorders; from predictive biomarkers to minimally invasive techniques

**DOI:** 10.3389/fsurg.2024.1487878

**Published:** 2024-10-14

**Authors:** M. P. Dimopoulos, G. I. Verras, F. Mulita

**Affiliations:** ^1^ Department of Radiology, General University Hospital of Patras, Patras, Greece; ^2^ Department of General Surgery, University Hospital Southampton, National Health Service (NHS) Trust, Southampton, United Kingdom; ^3^ Department of Surgery, General University Hospital of Patras, Patras, Greece; ^4^ Department of Surgery, General Hospital of Eastern Achaia- Unit of Aigio, Aigio, Greece

**Keywords:** colorectal cancer, colorectal surgery, colorectal surgery complications, biomarker, butyrylcholinesterase (BChE), postoperative complications

**Editorial on the Research Topic**
Newest challenges and advances in the treatment of colorectal disorders; from predictive biomarkers to minimally invasive techniques

The latest issue of Frontiers in Surgery highlights significant strides in colorectal disorders research, with an array of studies examining novel prognostic tools, surgical techniques, and treatment strategies. These studies collectively underscore the importance of personalized medicine, offering new insights into predictive markers, surgical innovations, and the nuanced role of adjuvant therapies.

One study by Zheng et al. provides a detailed comparative analysis of laparoscopic-assisted transanal natural orifice specimen extraction (NOSE) vs. conventional laparoscopic surgery (CLS) for sigmoid and rectal cancer. Among 121 patients, NOSE was associated with a shorter total incision length, highlighting its cosmetic advantage. However, this benefit was offset by a longer operation time compared to CLS. Importantly, there were no significant differences in postoperative complications, such as bacterial culture positivity, intra-abdominal infections, or anastomotic leakage, nor were there differences in overall survival (OS) and disease-free survival (DFS) outcomes between the two groups. The findings suggest that while NOSE may be particularly suitable for patients who prioritize cosmetic outcomes, the extended operative duration warrants careful consideration in clinical decision-making.

In another significant contribution, Ying et al. conducted a systematic review and meta-analysis to assess the impact of adjuvant chemotherapy (ACT) on survival outcomes in node-negative CRC patients, with a focus on the presence of perineural invasion (PNI). Their analysis revealed that ACT significantly improved OS and DFS in patients with PNI, with hazard ratios (HR) of 0.52 and 0.53, respectively. However, ACT did not significantly affect DFS in patients without PNI. These findings underscore the potential of ACT as a particularly beneficial intervention for patients with PNI, while also suggesting that it may confer some survival advantage for those without PNI.

Colorectal cancer (CRC) is characterized by significant genetic, anatomical, and transcriptional diversity. The tumor microenvironment (TME) plays a critical role in CRC prognosis and treatment outcomes. It consists of various cellular components like cancer-associated fibroblasts, tumor-associated macrophages, and regulatory *T* cells, as well as extracellular elements that contribute to therapeutic resistance through mechanisms such as fibrosis and enzymatic degradation. Given its influence on therapy efficacy, the TME presents a promising area for drug discovery, with ongoing research focused on targeting TME components to improve CRC treatment strategies (1).

Colorectal cancer (CRC) with the BRAF V600E mutation is aggressive and resistant to conventional therapies, largely due to the enhanced MAPK pathway activation. Although MAPK inhibitors have shown limited clinical success, combining these inhibitors with immune checkpoint inhibitors (ICIs) offers promise, particularly for patients with microsatellite instability-high (MSI-H) tumors (2).

The study by Jiang et al. investigated the relationship between collagen structure and the Immunoscore in the tumor microenvironment (TME) of CRC Using multiphoton imaging, they developed a collagen signature from 327 stage I-III CRC patients, which was strongly correlated with the Immunoscore. A collagen nomogram was subsequently constructed, integrating the collagen signature with clinicopathological predictors. This nomogram demonstrated high predictive accuracy for prognosis, particularly in high-risk stage II and III patients, and was shown to be a valuable tool in identifying patients who might benefit most from adjuvant chemotherapy. This study highlights the potential of collagen structure as a biomarker for immunological activity within the TME, offering a novel approach to prognosis in CRC.

Zhao et al. focused on patients with pT4M0 colon adenocarcinoma (COAD), analyzing optimal treatment strategies using data from the SEER database. Their study, which included 8,843 patients, revealed that those who received surgery combined with postoperative adjuvant therapy had significantly better 3-year OS and cancer-specific survival (CSS) rates compared to those who underwent surgery alone. A nomogram was developed, incorporating variables such as age, race, N stage, serum CEA levels, tumor differentiation, and the number of resected lymph nodes, demonstrating strong predictive accuracy. This study emphasizes the importance of integrating surgery with adjuvant chemoradiotherapy in improving long-term survival in high-risk COAD patients.

In the realm of imaging, Bai et al. investigated the utility of contrast-enhanced ultrasound (CEUS) in evaluating the response to neoadjuvant chemoradiotherapy in locally advanced rectal cancer (LARC). Their retrospective study of 83 patients found that certain CEUS parameters, such as peak intensity (PI) and area under the curve (AUC), were significantly associated with clinical outcomes. Patients with higher PI and AUC values, along with poorly differentiated tumors, had worse overall survival (OS) and progression-free survival (PFS). This study highlights the potential of CEUS quantitative analysis as a non-invasive tool for predicting treatment response and prognosis in LARC patients.

Zhong et al. examined predictive factors for achieving a pathologic complete response (pCR) in LARC patients treated with neoadjuvant chemoradiation (nCRT). Their analysis identified gross tumor volume (GTV) and tumor differentiation as significant predictors of pCR, with a tumor volume threshold of 21.1 cm³ showing a high sensitivity for predicting pCR. These findings suggest that GTV and tumor differentiation are crucial in preoperative assessments, helping clinicians in tailoring treatment plans more effectively.

Gallo et al. reported that, minimally invasive techniques such as laparoscopic surgery and SILS have transformed colorectal surgery, significantly improving patient outcomes by reducing recovery times and hospital stays. Emerging technologies, including robotic platforms and AI integration, are further enhancing surgical precision, setting the stage for future advancements in colorectal care (3).

A retrospective multicenter study, analyzing 5,398 rectal cancer surgeries, identified independent risk factors for anastomotic leakage, including sex, BMI, tumor location, and surgical approach (4). The overall leak incidence was 10.2%, with a 2.6% 30-day leak-related mortality. While protective stomas did not reduce leakage rates, they effectively minimized the severity and need for reoperation. The study introduced a clinical prediction model, the RALAR score, to assess individual risk and guide decisions on stoma construction post-resection. These findings may assist in optimizing surgical planning and patient outcomes.

A study by Zhao et al. found that the pan-immune-inflammation value (PIV) is significantly associated with tumor stage and other clinicopathological features in colorectal cancer (CRC) patients, showing potential as a preoperative assessment tool (5). PIV, particularly when combined with markers like CEA and CA19-9, demonstrated better predictive efficacy for CRC staging compared to other immune-inflammatory biomarkers.

Lastly, Verras and Mulita explored the potential of butyrylcholinesterase (BChE) as a predictive biomarker for surgical site infections (SSIs) after colorectal surgery (6). Their prospective study found that low BChE levels on the first and third postoperative days were associated with a significantly higher risk of SSIs. This suggests that BChE could serve as a valuable early marker for identifying patients at increased risk of infection, thereby enabling more timely and targeted interventions.

Benign colorectal diseases include a variety of conditions such as adenomatous polyps, diverticular disease, and inflammatory bowel disease (IBD) (7). These conditions pose significant challenges in clinical management, particularly in accurately identifying which lesions require intervention and which can be monitored safely. Over-diagnosis can lead to unnecessary interventions, increasing patient anxiety, complications and healthcare costs. Additionally, managing complications associated with these diseases, like diverticulitis, adds to the complexity. Future directions should focus on improving screening techniques, incorporating advanced imaging and molecular diagnostics for better characterization of lesions. Research into novel biomarkers for early detection and risk stratification in patients with IBD may also lead to more personalized treatment approaches.

In daily clinical practice, findings from recent studies can enhance decision-making by improving risk assessments and tailoring treatments. For instance, the pan-immune-inflammation value (PIV) can serve as a useful preoperative marker to assess tumor progression in colorectal cancer. The RALAR score aids in identifying patients at higher risk of anastomotic leakage after rectal surgery, guiding stoma construction decisions. Additionally, early postoperative butyrylcholinesterase (BChE) levels can help predict the risk of surgical site infections, enabling timely interventions and improved patient outcomes.

In conclusion, these studies collectively advance our understanding of CRC management, offering new prognostic tools and insights into the effectiveness of various treatment strategies. As personalized medicine continues to evolve, the integration of novel biomarkers, imaging techniques, and tailored therapeutic approaches will be essential in improving patient outcomes in colorectal disorders.
